# Licochalcone A improves the cognitive ability of mice by regulating T- and B-cell proliferation

**DOI:** 10.18632/aging.202704

**Published:** 2021-03-10

**Authors:** Yating Wu, Jianbo Zhu, Haifeng Liu, Hailiang Liu

**Affiliations:** 1 Key Laboratory of Xinjiang Phytomedicine Resource and Utilization of Ministry of Education, College of Life Sciences, Shihezi University, Shihezi 832003, China; 2 Institute for Regenerative Medicine, Shanghai East Hospital, Tongji University School of Medicine, Shanghai 200123, China; 3 China Colored-Cotton (Group) Co., Ltd., Urumqi 830016, Xinjiang, China

**Keywords:** licochalcone A, cognitive ability, T cells, B cells

## Abstract

Licochalcone A (LA), a flavonoid found in licorice, has anticancer, antioxidant, anti-inflammatory, and neuroprotective properties. Here, we explored the effect of injecting LA into the tail vein of middle-aged C57BL/6 mice on their cognitive ability as measured by the Morris water maze (MWM) test and cerebral blood flow (CBF). The related mechanisms were assessed via RNA-seq, and T (CD3e^+^) and B (CD45R/B220^+^) cells in the spleen and whole blood were quantified via flow cytometry. LA improved the cognitive ability, according to the MWM test results, and upregulated the CBF level of treated mice. The RNA-seq results indicate that LA affected the interleukin (IL)-17 signaling pathway, which is related to T- and B-cell proliferation, and the flow cytometry data suggest that LA promoted T- and B-cell proliferation in the spleen and whole blood. We also performed immune reconstruction via a tail vein injection of lymphocytes into B-NDG (NOD-*Prkdc*^scid^*Il2rg*^tm1^/Bcge) mice before treating them with LA. We tested cognitive ability by subjecting these animals to new object recognition tests and quantified the splenic and whole blood T and B cells. Cognitive ability improved after immune reconstruction and LA treatment, and LA promoted T- and B-cell proliferation in the spleen and whole blood. This study demonstrates that LA, by activating the IL-17 signaling pathway, promotes T- and B-cell proliferation in the spleen and whole blood of mice and improves cognitive ability. Thus, LA may have immune-modulating therapeutic potential for improving cognition.

## INTRODUCTION

Cognitive decline is a characteristic of human aging, but age-related learning and memory deterioration also occur in rats and mice [1–3]. Aging is regulated by a variety of systems in the body, such as the nervous system [4], immune system [5], and metabolic system.

The immune system modulates the population sizes and functions of various cell subsets; this system can also increase inflammation and contribute to immune senescence [6]. T cells play a key role in the immune system. For example, regulatory T cells (Tregs) regulate oligodendrocyte differentiation, and central nervous system function is also affected by Tregs [7, 8]. Whether changes in the immune system can affect cognition still needs further research.

**Figure 1:**
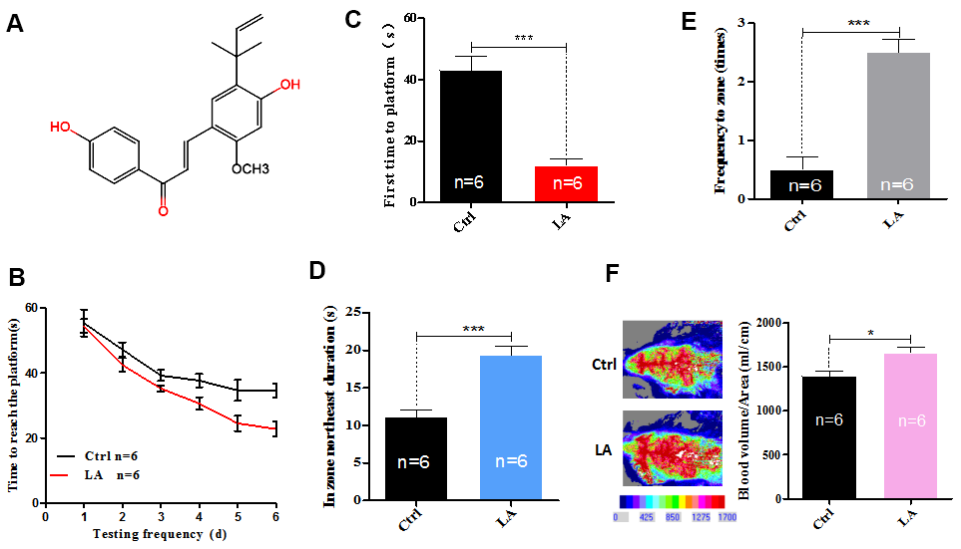
**Effect of LA treatment on the cognitive ability of middle-aged C57BL/6 mice.** (**A**) The chemical structure of LA. (**B**) Time taken by LA-treated or control (Ctrl)-treated mice to reach the platform in the MWM test over a 6-day experiment. (**C**) First latency to the platform in the MWM test. (**D**) In zone target duration in the MWM test. (**E**) Frequency in zone in the MWM test. (**F**) Cerebral blood flow level in LA- or Ctrl-treated mice. The data are presented as the mean±SD of three independent experiments. Statistical significance was determined using unpaired *t*-tests. *p < 0.05, **p < 0.01 compared with the Ctrl group; “n” indicates the number of animals in each experimental group.

Licochalcone A (LA) is a flavonoid found in licorice (structure shown in Figure 1) that has been shown to have various clinically interesting pharmacologic effects, including antioxidant [9], anti-inflammatory [10], anticancer [11], and neuroprotective effects [12]. However, the mechanisms by which LA affects the immune system and the cognitive consequences of these effects are unknown. Here, to determine if LA has potential for development as a therapeutic treatment to improve cognitive ability, we first tested whether LA could improve the cognitive abilities of mice and investigated the underlying mechanisms by conducting RNA-seq of the hippocampus and assessing the proliferation of lymphocytes, such as T cells, B cells, and natural killer (NK) cells in the spleen and whole blood. We also treated immune-reconstructed B-NDG (NOD-*Prkdc*^scid^*Il2rg*^tm1^/Bcge) mice with LA, then subjected the animals to new object recognition tests and measured the T- and B-cell levels in the spleen and whole blood.

## RESULTS

### LA improves the cognitive ability of middle-aged C57BL/6 mice

Cognitive ability declines with age [13, 14], and the Morris water maze (MWM) test [15] is commonly used to assess the spatial memory and cognitive ability of aging mice. In the present study, middle-aged mice were injected with LA into the tail vein every other day for a month and then subjected to the MWM test. The results show that, over the course of 6 days, the time that it took for the LA-treated mice to find the platform gradually decreased, unlike that in the control-treated mice (Figure 1B). The lower amounts of time required for the LA-treated mice to find the platform suggest that treatment with LA improved the spatial memory and cognitive ability of middle-aged C57BL/6 mice. To further evaluate the spatial memory and cognitive ability of the mice, analysis software was used to analyze their swimming tracks over 1 min, and the following three indicators were included: first time to platform (Figure 1C), in zone target duration (Figure 1D), and frequency in zone (Figure 1E). The MWM test results show that LA treatment significantly improved all measured indices related to cognitive ability in middle-aged C57BL/6 mice compared with control treatment.

Cerebral blood flow (CBF) is a key factor related to cognitive ability [16], and when the level of CBF is depressed, it can lead to cognitive dysfunction [17]. Changes in CBF are associated with various brain disorders [18]. CBF in the occipital lobe is important for brain functioning and regulates multiple neurological processes [19]. Because changes in the CBF are related to cognitive impairment [20], we measured the CBF in the LA-treated and control-treated mice. The results show that the CBF level in middle-aged C57BL/6 mice was higher for LA-treated animals than it was for control-treated animals (Figure 1F). The MWM test and CBF results indicate that LA treatment improves the cognitive ability of middle-aged C57BL/6 mice.

### LA affects the immune system by regulating the interleukin (IL)-17 signaling pathway

**Figure 2:**
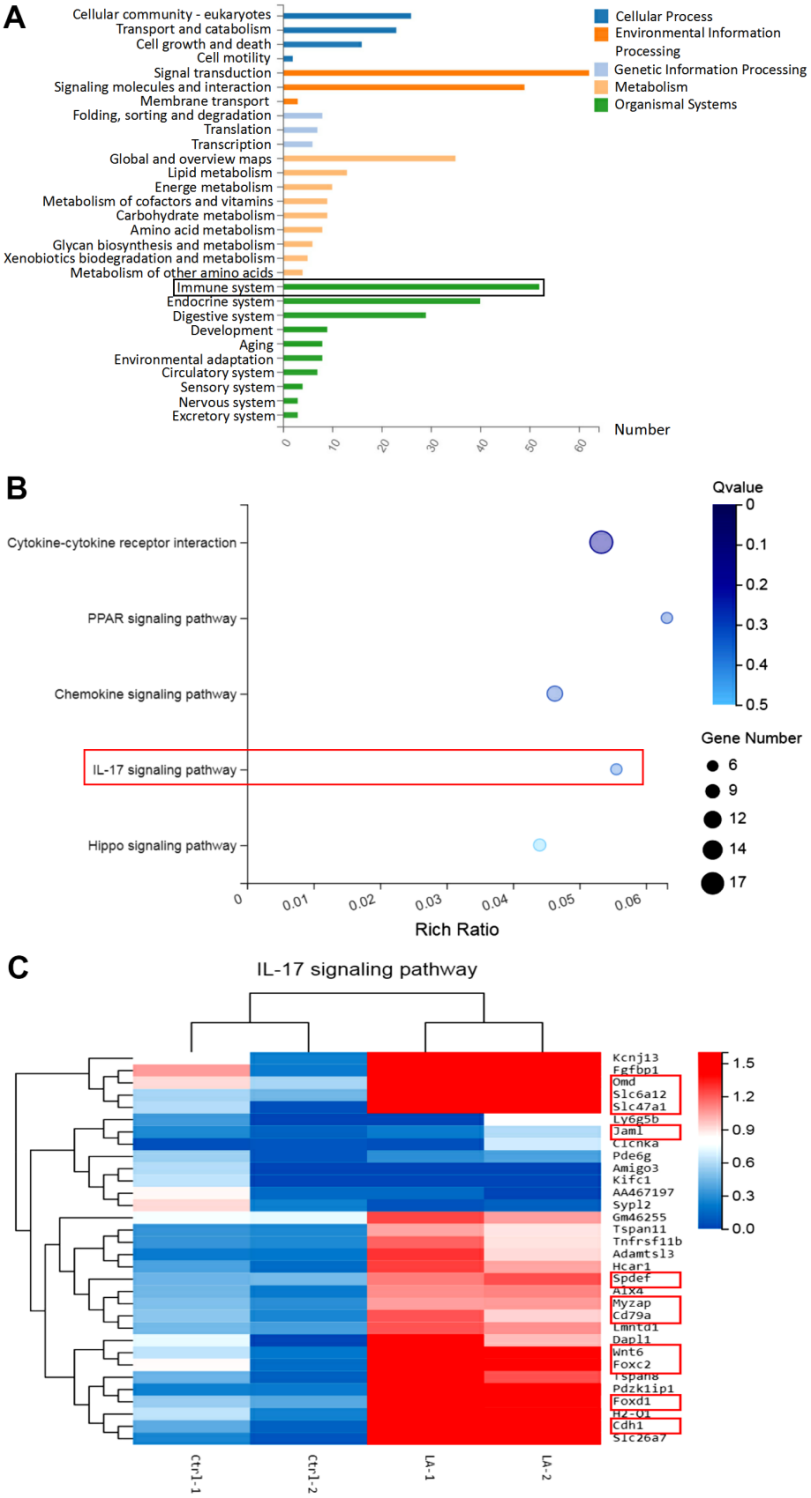
**Analysis of the mechanism by which LA regulates the immune system conducted using RNA-seq in the hippocampus.** (**A**) Kegg pathway classification map. (**B**) Bubble diagram showing the pathways regulated by LA. (**C**) Heatmap showing the differentially expressed genes related to the IL-17 signaling pathway. The data are presented as the mean±SD of three independent experiments. Statistical significance was determined using unpaired *t*-tests. *p < 0.05, **p < 0.01 compared with the Ctrl group.

Because the behavioral experiment results suggested that LA treatment improves cognitive ability, we next investigated the associated mechanism by performing RNA-seq on the hippocampus of C57BL/6 mice. The resulting Kyoto Encyclopedia of Genes and Genomes (KEGG) pathway classification map, shown in Figure 2A, detected that 52 signaling pathways were found to be associated with the immune system. As seen in the bubble diagram shown in Figure 2B, LA was found to regulate the interleukin (IL)-17 signaling pathway, which is important in immune system function [21, 22]. Furthermore, as seen in the heatmap of differentially expressed genes related to the IL-17 signaling pathway, 11 of the detected genes are related to the proliferation of lymphocytes, including T cells, B cells, and NK cells (Figure 2C and Table 1) [23–45]. The RNA-seq analysis results suggest that LA treatment affects the proliferation of lymphocytes, including T cells and B cells, by regulating the IL-17 signaling pathway.

**Table 1:** Table for genes in heatmap which regulate lymphocytes proliferation.

**Genes regulated by IL-17 signaling pathway which related to lymphocytes**			
**Genes**	**Lymphocytes**	**Genes**	**Lymphocytes**
Omd	T cell [23], B cell [24], NK cell [25]	cd79a	T cell [36], B cell [37]
Slc6a12	T cell [26], B cell [27], NK cell [28]	Wnt6	T cell [38], B cell [39]
Slc47a1	T cell [29], B cell [30], NK cell [31]	Foxc2	T cell [40], B cell [41]
Jaml	T cell [32]	Foxd1	T cell [42], B cell [43]
Myzap	T cell and B cell [33]	Cdh1	T cell [44], B cell [45]
Spdef	T cell [34], NK cell [35]		

### LA promotes T- and B-cell proliferation in middle-aged C57BL/6 mice

**Figure 3:**
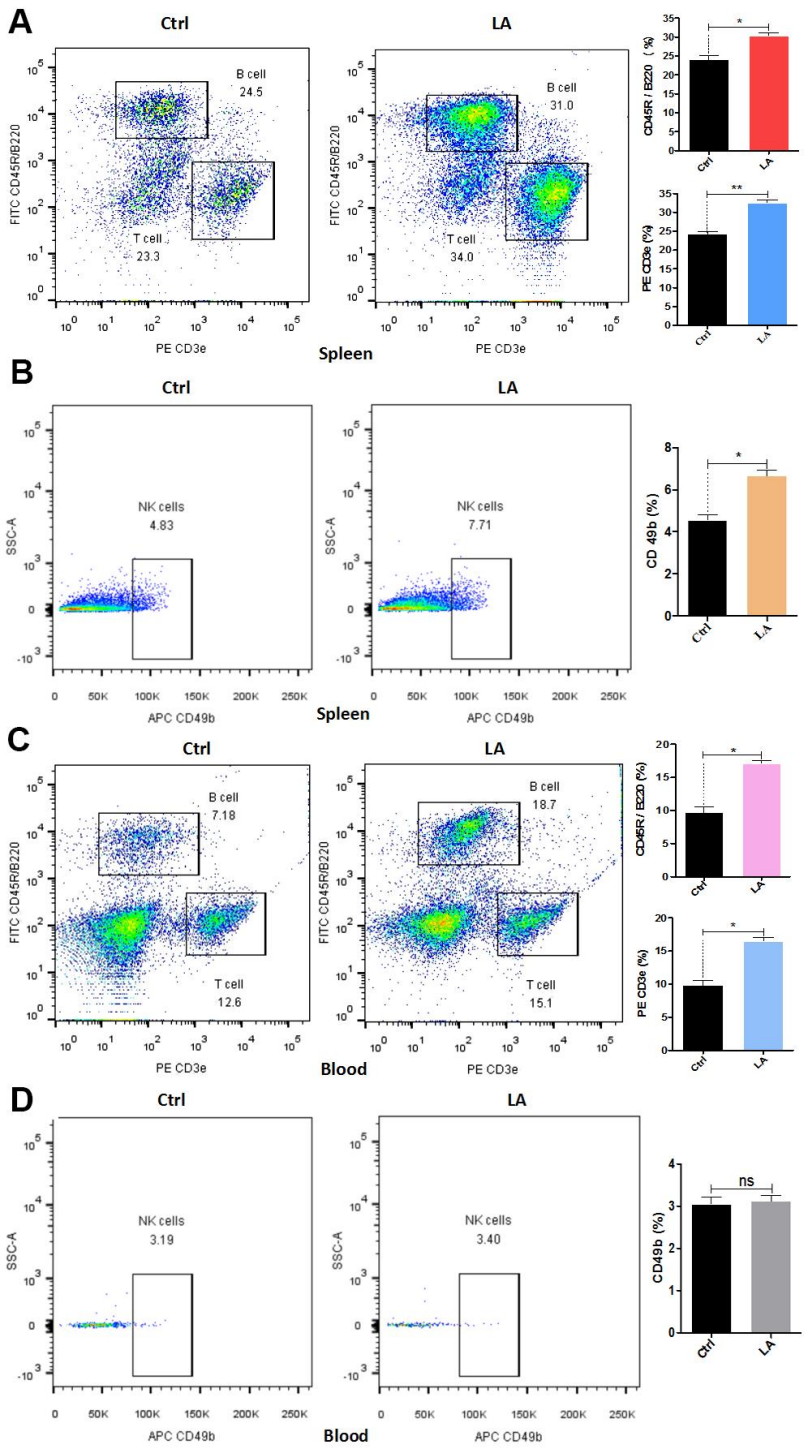
**LA effect on lymphocyte proliferation in C57BL/6 mice.** (**A**–**D**) T-cell and B-cell (**A**, **C**) or NK-cell (**B**, **D**) proliferation in the spleen (**A**, **B**) or whole blood (**C**, **D**) of LA- or control (Ctrl)-treated mice. The data are presented as the mean±SD of three independent experiments. Statistical significance was determined using unpaired *t*-tests. *p < 0.05, **p < 0.01 compared with the Ctrl group.

To determine whether LA affects lymphocyte proliferation, the percentages of T cells, effector T cells, B cells, effector B cells, and NK cells among the lymphocytes in the spleen and whole blood of LA- or control-treated middle-aged C57BL/6 mice were measured by flow cytometry. In the spleen, the percentages of B cells (CD45R/B220^+^), T cells (CD3e^+^), and NK cells (CD49b^+^) were all significantly higher in the LA-treated mice compared with the control-treated mice (Figure 3A, 3B). Similarly, the percentage of effector T cells (CD44^+^) in the spleen was significantly higher in the LA-treated mice than in the control-treated mice (Supplementary Figure 1A, 1C); however, the percentage of effector B cells (CD27^+^) was not different between the two treatment groups (Supplementary Figure 1B, 1D).

As in the spleen, the percentages of B cells, T cells, and effector T cells in whole blood were all significantly higher for the LA-treated mice than for the control-treated mice (Figure 3C and Supplementary Figure 1A), whereas the percentage of effector B cells was not different between the two groups. However, unlike in the spleen, the percentage of NK cells also did not differ between the two treatment groups (Figure 2D).

*In vitro* experiments were conducted to evaluate the effect of LA on T-cell and B-cell proliferation. The results show that immune cells from the spleens of LA-treated mice promoted significantly more T-cell and B-cell proliferation compared with those from control-treated mice (Supplementary Figure 2A). The flow cytometry results reveal that immune cells from the blood of LA-treated mice also promoted T-cell and B-cell proliferation significantly more compared with blood immune cells from control-treated mice (Supplementary Figure 2B). The relative mRNA expression results show that the mRNA expression of T cells (CD3e^+^) and B cells (CD45R/B220^+^) was increased by LA treatment as compared with control treatment (Supplementary Figure 2C).

The flow cytometry results from *in vitro* experiments suggest that LA treatment promoted the proliferation of T and B cells. The expression of homeobox B (*HOXB*) cluster genes was evaluated as a measure of activated lymphocytes [46] because *HOXB*-3 plays an essential role in regulating B lymphopoiesis in mice [47] and *HOXB*-3 overexpression promotes the proliferation and differentiation processes of lymphocytes [48], while *HOXB*-5 is dispensable for T-cell proliferation [49]. The runt-related transcription factor (*RUNX*) family is involved in the development of T and B lymphocytes [50]; *RUNX*-1 functions in T-cell population maintenance [51], and *RUNX*-2 can promote B-cell proliferation [52]. Additionally, T and B cells are also influenced by *RUNX*-3 [53]. To learn about the mechanism of how LA activates the immune system *in vitro*, we examined the mRNA expression of *in vitro* spleen cells. The results show that in LA-treated cells, the mRNA expression levels of *HOXB*-3, *RUNX*-2, and *RUNX*-3 are all significantly higher than those in control-treated cells (Supplementary Figure 2D). Thus, the mechanism by which LA activates the immune system, at least *in vitro*, involves the activation of *HOXB*-3, *RUNX*-2, and *RUNX*-3 expression to promote the proliferation of T and B cells.

To further investigate the effect of LA on neurons, we examined expression of the neural stem cell marker SOX2 [54, 55] in the hippocampus and subventricular zone (SVZ) of LA-treated mice and control mice. The results show that *SOX*2 expression in the hippocampus was significantly higher for the LA-treated aging mice than for the control-treated aging mice (Supplementary Figure 2E, 2F). The result also indicates that LA treatment improved the cognition of middle-aged C57BL/6 mice.

In conclusion, LA promotes the proliferation of T and B cells in middle-aged C57BL/6 mice by activating the IL-17 signaling pathway, and the results of *in vitro* work suggest that LA treatment improves the percentage of T and B cells via activating the expression of *HOXB*-3, *RUNX*-2, and *RUNX*-3.

### LA treatment can promote T- and B-cell proliferation in B-NDG mice

The experiments conducted in C57BL/6 mice described above revealed that LA treatment improved the T- and B-cell proliferation in the whole blood and spleen. To further investigate the relationship between LA treatment and the immune system, we next conducted experiments in B-NDG mice, which have a severe immune defect phenotype [56]. The B-NDG mice underwent immune reconstitution (IR) via a tail vein injection of lymphocytes, after which they were treated with LA for 30 days.

**Figure 4:**
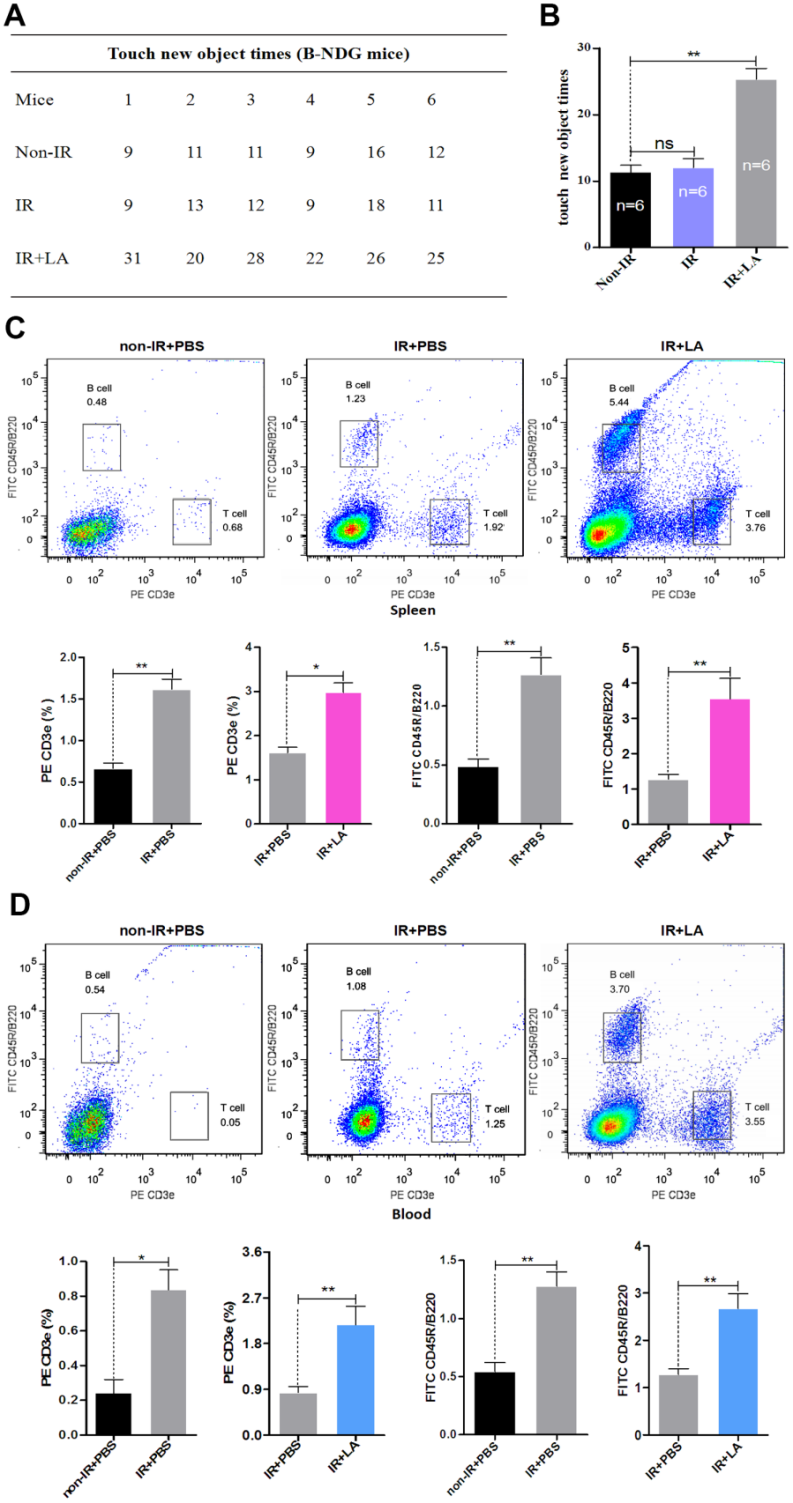
**Effect of LA on T- and B-cell proliferation in B-NDG mice.** (**A**) New object recognition results for the IR+LA, IR and non-IR groups. (**B**) Statistical analysis of the results shown in (**A**). (**C**, **D**) T- and B-cell proliferation in the spleen (**C**) and whole blood (**D**). The data are presented as the mean±SD of three independent experiments. Statistical significance was determined using unpaired *t*-tests. *p < 0.05, **p < 0.01 compared with the non-IR group; “n” indicates the number of animals for each experimental group.

Previous work found that meningeal T-cell composition is coupled to the central nervous system draining deep cervical lymph nodes, that the normal flow of meningeal T cells regulates cognition [57], and that an increase in the percentage of circulating B and T cells ameliorated brain cognition [58]. Because cognitive ability has been reported to be partially regulated by the immune system [59, 60], we tested the effect of LA treatment on the cognitive ability of B-NDG mice by using the new object recognition test. The new object-touch time for LA-treated IR mice was significantly higher than that for PBS-treated IR mice and PBS-treated non-IR mice (Figure 4A, 4B). These results suggest that LA treatment improved the memory of B-NDG mice. Prior research suggested that T lymphocytes contribute to an increase in hippocampal neurogenesis and working memory [61] and that B lymphocytes may represent a therapeutic option for the treatment of cerebral contusion [62]. These findings indicate that memory improvement is closely related to the regulation of T and B cells.

Because our above results suggested that LA treatment promotes T- and B-cell proliferation, the improved memory of LA-treated IR B-NDG mice may be due to an LA treatment-induced increase in T and B cells. To further investigate this possibility, we examined the lymphocyte populations in B-NDG mice. The percentages of T cells and B cells in the spleen were significantly higher in LA-treated IR mice than in PBS-treated IR mice or PBS-treated non-IR mice (Figure 4C). However, in whole blood, the percentage of T cells was significantly higher in LA-treated IR mice than in PBS-treated IR mice or PBS-treated non-IR mice, and the percentage of B cells in these mice was significantly higher compared with that in the PBS-treated IR mice or PBS-treated non-IR mice (Figure 4D). These results suggest that LA treatment in B-NDG mice promotes the production of lymphocytes, specifically T cells and B cells.

Because the behavioral experiment and flow cytometry results suggested that LA treatment improves the cognitive ability of IR mice, we next investigated the associated mechanism by performing RNA-seq on the hippocampus of B-NDG mice. The differentially expressed genes between the PBS-treated IR mice and PBS-treated non-IR mice, PBS-treated IR mice and LA-treated IR mice, and PBS-treated non-IR mice and LA-treated mice are listed in Supplementary Figure 3A. In the LA-treated mice, 126 of the differentially expressed genes were upregulated and 26 of these genes were downregulated. As shown by the bubble diagram in Supplementary Figure 3B, LA was found to regulate the Th1- and Th2-cell differentiation signaling pathway, which controls Th1- and Th2-cell differentiation during the T-cell activation process [63]; notably, Th1 and Th2 are involved in the pathogenesis of many immune-mediated diseases [64]. Furthermore, as seen in the heatmap shown in Supplementary Figure 3C, among the differentially expressed genes related to the Th1- and Th2-cell differentiation signaling pathway, the H2-Eb1, H2-Ab1, H2-Aa, CD74 genes were upregulated in the LA-treated IR mice as compared with their levels in the PBS-treated IR mice or PBS-treated non-IR mice. Previous work suggested that the activation of H2-Eb1, H2-Ab1, H2-Aa, and CD74 expression promotes T-cell synapse formation [65]. Additionally, another study reported that T cells were significantly higher among autoantibodies against CD74 [66], and anti-CD74 monoclonal antibody may provide a basis for novel therapeutic approaches to treating B-cell malignancies [67]. Furthermore, we conducted a gene set enrichment analysis of these differentially expressed genes, which similarly revealed that LA treatment activated the Th1- and Th2-cell differentiation signaling pathway (Supplementary Figure 3D). The RNA-seq analysis results suggest that LA treatment affects the proliferation of lymphocytes, including T cells and B cells, by activating the Th1- and Th2-cell differentiation signaling pathway.

*In vitro* experiments were conducted to evaluate the effect of LA on the proliferation of T or B cells. The results show that immune cells from the spleens of IR mice treated with LA promoted the proliferation of T and B cells significantly more as compared with splenic immune cells from PBS-treated IR mice (Supplementary Figure 4A). The flow cytometry results reveal that immune cells from the blood of LA-treated IR mice also promoted the proliferation of T cell significantly more as compared with blood immune cells from PBS-treated IR mice, whereas the percentage of B cells did not differ significantly between the two groups (Supplementary Figure 4B). The relative mRNA expression results show that the mRNA expression of T cells (CD3e^+^) and B cells (CD45R/B220^+^) is increased by LA treatment (Supplementary Figure 4C).

As shown above, the *in vitro* flow cytometry results indicated that LA treatment promotes T-cell and B-cell proliferation. To learn about the mechanism by which LA activates the immune system *in vitro*, we next examined the mRNA expression of *in vitro* spleen cells. The mRNA expression levels of *HOXB*-2, *HOXB*-3, and *RUNX*-2 were significantly higher in LA-treated IR cells than in PBS-treated IR cells (Supplementary Figure 4D). Thus, the mechanism by which LA activates the immune system *in vitro* involves the activation of *HOXB*-2, *HOXB*-3, and *RUNX*-2 expression, which promote the proliferation of T and B cells.

In conclusion, LA can activate the immune reconstitution of B-NDG mice by promoting the proliferation of T and B cells, and *in vitro* experiments further revealed that LA promotes T-cell proliferation by activating the expression of *HOXB*-2, *HOXB*-3, and *RUNX*-2.

## DISCUSSION

Here, to investigate whether LA can improve cognitive ability, LA was injected into the tail veins of middle-aged mice, and the animals were assessed by using the MWM test, which measures cognitive ability [68], and measuring their CBF levels, which are a key factor for neuroprotection [69]. The MWM test results indicate that LA improved the memory of treated mice, and the CBF results show that LA treatment upregulates the CBF level.

RNA-seq was performed on hippocampus samples; the results show that LA treatment affected the IL-17 signaling pathway and that the signal pathway changes were related to the proliferation of lymphocyte subsets, including T cells, B cells, and NK cells, through the regulation of immune system gene expression. IL-17 is a proinflammatory cytokine that is crucial for a variety of processes, including host defense and inflammatory disease pathogenesis [70]. Besides having proinflammatory roles, IL-17 is also a neuromodulator [71], and the IL-17 signaling pathway regulates multiple phenotypes, including learning, immunity, and longevity [72].

Our flow cytometry results indicate that LA treatment significantly promoted the proliferation of T cells and B cells in the spleen and whole blood. Previous work found that IL-17 can regulate T-cell proliferation in the peripheral blood [73]. Additionally, IL-17 can activate B cells, which leads to an inflammatory immune reaction [74], and can promote T cell–B cell interactions that induce B-cell expansion and antibody production [75].

Together, our findings in middle-aged C57BL/6 mice indicate that LA promotes T- and B-cell proliferation in the spleen and whole blood by regulating the IL-17 signaling pathway. Our *in vitro* results further suggest that LA treatment promotes the proliferation of T and B cells by activating the expression of *HOXB*-3, *RUNX*-2 and *RUNX*-3.

The results of our new object recognition experiment in LA-treated IR B-NDG mice provide further evidence that LA treatment improves the cognitive ability of mice. Additionally, the flow cytometry results show that LA promoted T- and B-cell proliferation in the spleen and promoted T-cell proliferation in the blood of these animals. Thus, LA may improve the cognitive ability of mice by stimulating the proliferation of lymphocytes, such as T cells and B cells. Other research in B-NDG mice, which are severely immunodeficient mice that do not have lymphocytes such as mature T or B cells, found that treatment targeting chimeric antigen receptor (CAR)-modified T cells promotes T-cell proliferation [76], which further supports our finding that LA treatment improves T- and B-cell proliferation. The results of our *in vitro* experiments also suggest that LA treatment promotes the proliferation of T cells by activating the expression of *HOXB*-2, *HOXB*-3, and *RUNX*-2 genes.

Our results indicate that LA treatment promotes T- and B-cell proliferation and improves the cognitive ability of middle-aged C57BL/6 mice by activating the IL-17 signaling pathway. Previous research in rats revealed that increasing the Th1/Th2 cytokine balance in the hippocampus reduced cognitive deficits by improving memory [77]. Lymphocyte activation has a clinical impact, as indicated by the reported correlation between activated T-cell levels and the pathology of Alzheimer’s disease [78], and B-cell diapedesis, which mediated memory recovery, occurred in areas remote to the infarction [79]. Thus, T- and B-cell proliferation play an important role in improving memory by regulating related signaling pathways.

LA, a major flavonoid in *Glycyrrhiza inflata*, has multiple known pharmacological effects [80]; here, we explored the influence of LA on cognitive ability from the perspective of the immune system. *G. inflata* is an important Chinese medicinal plant and is considered to have potential for future drug development as an anti-coronavirus 19 agent [81, 82]. There are many molecules known to be capable of promoting lymphocyte proliferation. For example, icariin exerts neuroprotective effects via modulating the CD4^+^ T lymphocyte-related immunoinflammatory responses in APP/PS1 mice and may be a promising drug against Alzheimer’s disease progression [83]. Additionally, glycyrrhizic acid also improves cognition in aging mice through promoting T- and B-cell proliferation [84], and resveratrol significantly improves the growth of T cells in the human circulating immune system [85]. Furthermore, ginsenoside Rg1 ameliorates aging through upregulating T-cell proliferation [86]. Thus, exploring the effects of active ingredients in medicinal plants can reveal clinically useful findings.

In conclusion, our data suggest that LA can improve cognitive ability by inducing T-cell proliferation in the spleen and whole blood. This is the first demonstration of LA affecting the immune function of middle-aged C57BL/6 mice and B-NDG mice. Additionally, LA was found to affect the IL-17 signaling pathway by regulating T- and B-cell proliferation in middle-aged C57BL/6 mice. Furthermore, LA was found to affect the Th1- and Th2-cell differentiation signaling pathway and promote IR by regulating T- and B-cell proliferation in B-NDG mice. These findings suggest that it may be possible to improve cognitive ability by activating the immune system via the use of a small molecular compound.

## MATERIALS AND METHODS

### Drug

LA (C_21_H_2_O_4_; MW: 338; purity ≥98%, HPLC grade) was purchased from Chengdu Push Biotechnology Co. Ltd. (Sichuan, China) and stored at 2-8° C in a dark, dry place. The stock solution had a concentration of 15 mg/L in phosphate-buffered saline (PBS).

### Animals

This study was approved by the Animal Research Committee of Tongji University School of Medicine and was conducted in accordance with institutional guidelines. For treatment, C57BL/6 mice (12 months old, female) purchased from Shanghai SLAC Laboratory Animal Co. were injected with LA via the tail vein every other day for a month. B-NDG mice (8 weeks old, female) purchased from Jiangsu Biocytogen Laboratory Animal Co. (BCM002F). All mice were maintained on a 12-h light/dark cycle with free access to food and water. Each experimental group was composed of six mice.

### MWM test

For the MWM test, a cylindrical tank with a diameter of 120 cm was filled with water. The water temperature was maintained at room temperature, and a platform was placed 1cm below the water level for use as a marker of mouse cognition. The spatial positions corresponding to the southeast (SE), east (E), southwest (SW), and west (W) directions of the platform were marked with different colors to provide a reference for spatial memory in the cognitive process of the mice.

Our experiment was divided into two stages. In the first stage, each mouse was required to swim for 1 min in the four directions corresponding to the platform in the pool. In the second stage, the platform was removed from the pool on day 6, and the mice were left to swim for 1 min at the farthest point from the platform. The swimming track of each mouse was recorded by a computerized tracking/image analyzer system [87].

### CBF detection

The brains of anesthetized mice were exposed, and images were acquired with a laser speckle contrast imager (PeriCam PSI System, Perimed, Stockholm, Sweden). The PeriCam PSI HD system was used to calculate an arbitrary index of cerebral blood flow (perfusion units) in the ipsilateral hemisphere.

### RNA-seq analysis

RNA-seq was performed independently and uniformly on the total RNA extracted from hippocampus samples from LA- or control-treated mice. The clean reads were aligned to the reference gene sequence using bowtie-2, and the gene expression levels of each sample were calculated. Differential gene detection was conducted by applying the DEGseq method [88]. The statistical results are based on the ma-plot method, in which the number of reads for specific genes obtained from the sample were randomly sampled, and then *p*-values were calculated according to the normal distribution and corrected to q-values. To improve the accuracy of identified differentially expressed genes (DEGs), genes with a difference multiple of more than double the lower value and a q-value of ≤0.001 were defined and screened as significant DEGs. RNA sequencing data were deposited at GSE144123.

### Flow cytometry

Single-cell suspensions were generated from the spleen and blood collected from experimental mice. After red blood cells were eliminated by using the Red Cell Lysis buffer solution (BD Biosciences, San Jose, CA, USA), lymphocytes were filtered through a 70-μm cell strainer (Jet Biofil, Guangzhou, China) and diluted in 1× PBS (Thermo Fisher Scientific, Waltham, MA, USA). T cells (CD3e^+^), B cells (CD3e^−^CD45R/B220^+^), NK cells (CD3e^−^CD49b^+^), effector T cells (CD45^+^CD3e^+^CD44^+^), and effector B cells (CD45^+^CD3e^−^, CD45R/B220^+^CD138^+^CD27^+^) in the spleen and blood were directly quantified using flow cytometry with monoclonal antibodies (1 μg/ml) directed against the noted specific human and mouse antigens (Table 2; all antibodies purchased from BD Pharmingen, San Diego, CA, USA). The stained cells were detected using a flow cytometer (Beckman FC-500, Miami, FL, USA) and analyzed with FlowJo 7.6.1. software.

**Table 2:** Antibodies for examining lymphocytes.

**Antibodies**	**Source**	**Article number**
CD45R/B220	BD Biosciences	Cat# 553087
CD3e	BD Biosciences	Cat# 553063
CD27	BD Biosciences	Cat# 558754
CD138	BD Biosciences	Cat# 558626
CD44	BD Biosciences	Cat# 553134
CD45	BD Biosciences	Cat# 566439
CD49b	BD Biosciences	Cat# 558295

### Immune reconstitution (IR)

Lymphocytes were harvested from middle-aged C57BL/6 mouse spleens by removing red blood cells with lysis buffer (BD Biosciences, San Jose, CA, USA) and filtering the cells through a 70-μm cell strainer (Jet Biofil, Guangzhou, China) using 1×PBS (Thermo Fisher Scientific, Waltham, MA, USA). The lymphocytes were kept alive by storing them on ice. These cells (3×10^5^ per animal) were then injected into the tail veins of 8-week-old B-NDG (NOD-*Prkdc*^scid^*Il2rg*^tm1^/Bcge) mice, after which the animals were treated with LA for 30 days.

### New object recognition test

The new object recognition test was employed to examine the memory ability of B-NDG mice [89]. This model evaluates the memory ability of the tested mice according to the length of exploration time spent on familiar objects (ones they have seen previously) and on new objects (ones they have not seen before). When the tested mice have not forgotten the familiar objects, they will spend more time exploring the new objects. However, when the tested mice have forgotten the familiar objects, they will spent roughly the same time exploring the new objects as they do exploring the familiar objects. VisuTrack of Animal Behavior software was used for the analysis.

### Cell culture

LA (C21H22O4; MW: 338; purity >98%, HPLC grade) was purchased from Chengdu Push Biotechnology Co. Ltd. (Sichuan, China) and stored at 2-8° C in a dark, dry place. The concentration of the stock solution was 100 mM in dimethyl sulfoxide (DMSO). The final DMSO concentration did not exceed 0.1% in the culture medium. Lymphocytes obtained from spleen and blood were cultured in a humidified 5% CO2 atmosphere at 37° C in DMEM/F12 (Thermo Fisher Scientific, Waltham, MA, USA) medium supplemented with 10% FBS and 100 U/mL penicillin and streptomycin.

### qRT-PCR analysis

Total RNA was isolated using TRIzol reagent (Thermo Fisher Scientific, Waltham, MA, USA), and cDNA was prepared using the Prime Script™ RT Master Mix (Perfect Real Time) (Takara, Dalian, China) according to the manufacturer’s protocols. The qRT-PCR reactions were performed using SYBR green fluorescent dye (BioRad). Primer sequences are listed in the Table 3.

**Table 3:** Primer sequences used in this study.

**Genes**	**Forward primers (5’-3’)**	**Reverse primers (5’-3’)**
**CD3e**	CTGCTACACACCAGCCTCAA	GTAATAAATGACCATCAGCAAGC
**CD45R**	CGGAAGTTCCTGGAGCACCTCTC	AAGTACACCTTGGCCCCCACGTA
**HOXB-2**	GATGGCCTGAACCTCATCGA	AGTTCGGTCCGGTTCCAGAT
**HOXB-3**	AGATATTCCCCTGGATGAAAGA	GAACTCCTTCTCCAG CTCCAC
**HOXB-5**	GTGCCAATGTTGTGTGTTGC	TCAGGTAGCTTGTTCCTTGG
**RUNX-1**	TGGCAGGCAACGATGAAAAC	CGCTCGGAAAAGGACAAACTC
**RUNX-2**	CAGGAAGAGCGGCAAGTATTA	AAGGTCCGAAGTTGAGGGAAA
**RUNX-3**	TTGCCAAGCCTTATCGGAA	CAGGGGAGAAATCGATGACA
**Actin**	TATTGGCAACGAGCGGTTC	ATGCCACAGGATTCCATACCC

### Immunofluorescence assay

From our experiment, mice from each of the LA group and control group were randomly selected. Following anesthesia and killing, half of the brain from each mouse was fixed with 4% paraformaldehyde (PFA) overnight at 4° C using 15% and 30% sucrose gradient dehydration. Next, optimal cutting temperature compound (OCT) (Tissue-Tek, USA) was used to coat the brain hemispheres at 4° C for 2 h to discharge air bubbles. Then the brains were placed in an embedding box in absolute ethanol at -80° C to allow slow solidification. Serial sections (sagittal section, 10 μm) were chosen per mouse by freezing microtome. The tissue slices were treated with methanol or 4% PFA for 10 min at -20° C. After three washes, all slides were incubated in blocking buffer (3% BSA 0.3% triton X-100 in PBS) for 1 h at room temperature. Slides were immersed in 200 μL primary anti-*SOX*2 antibody (1:500) per slide for overnight incubation at 4° C, followed by secondary antibody anti-rabbit for 1h in the dark at room temperature. DAPI reagent was used as a counter staining medium. The slides were examined using an Olympus BX53 microscope.

### Statistical analysis

Statistical analysis was performed using GraphPad Prism 5.0, and the data are expressed as the mean ± standard error of the mean (mean ± SD). Statistically significant differences between two groups were assessed by using unpaired *t*-tests. Statistical comparisons of more than two groups were performed through an analysis of variance (ANOVA). A two-tailed *p*-value of <0.05 was considered statistically significant.

## Supplementary Material

Supplementary Figures
