# Supplementary material for: Licochalcone A improves the cognitive ability of mice by regulating T- and B-cell proliferation
Source: Aging (Albany NY). 2021 Mar 10;13(6):8895–915. doi: 10.18632/aging.202704 (PMC8034954; doi:10.18632/aging.202704)
Supplement: Supplementary Figures [file aging-13-202704-s001.pdf]

SUPPLEMENTARY FIGURES

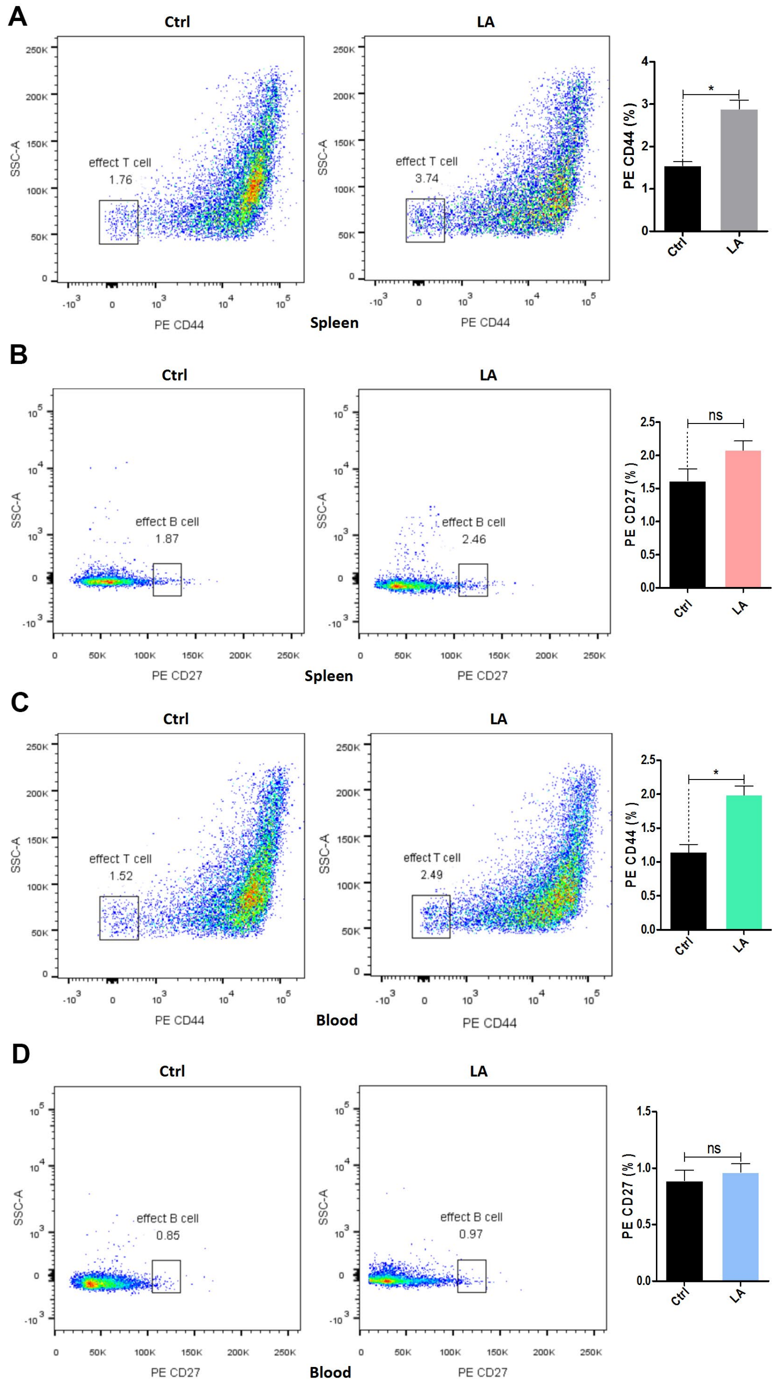

Supplementary Figure 1. LA effect on effector T-and B-cell proliferation in the spleen and whole blood in C57BL/6 mice. (**A**) Effector T-cell proliferation in the spleen of LA-or control (Ctrl)-treated mice. (**B**) Effector B-cell proliferation in the spleen of LA-or control (Ctrl)-treated mice. (**C**) Effector T-cell proliferation in the whole blood of LA-or control (Ctrl)-treated mice. (**D**) Effector B-cell proliferation in the whole blood of LA-or control (Ctrl)-treated mice. The data are presented as the mean±SD of three independent experiments. Statistical significance was determined using unpaired t-tests. $^*p < 0.05$, $^{**}p < 0.01$ compared with the Ctrl group.

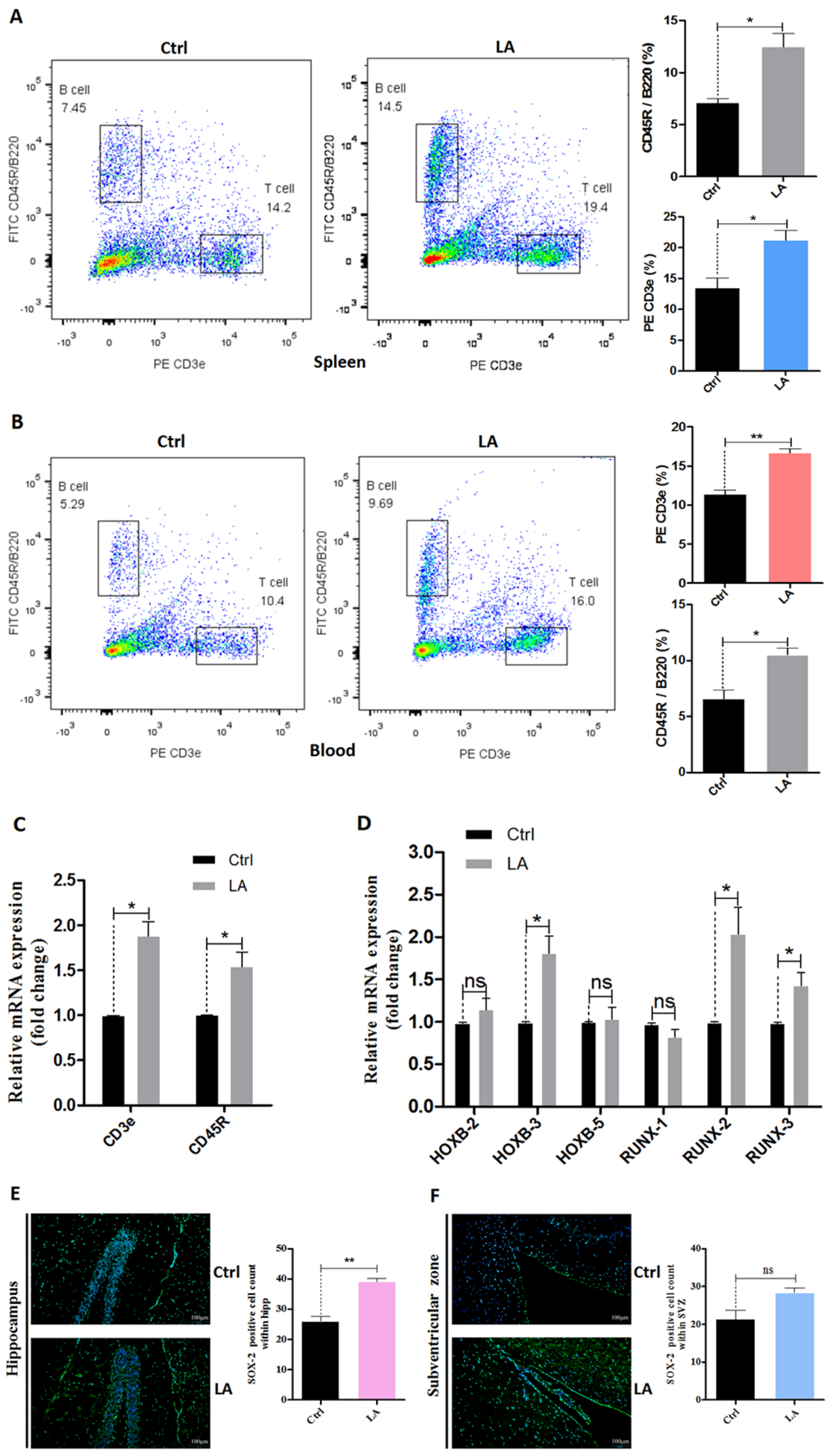

Supplementary Figure 2. Effect of LA on lymphocyte proliferation *in vitro*. (**A**, **B**) Proliferation of LA-treated or control (Ctrl)-treated *in vitro* cultured T and B cells from the spleen (**A**) or whole blood (**B**) of C57BL/6 mice. (**C**) Relative mRNA expression of *in vitro* splenic lymphocytes. (**D**) Relative mRNA expression of genes that might regulate lymphocyte proliferation. (**E**, **F**) Expression of the neural stem cell marker *SOX*-2 in the hippocampus (Hipp) (**E**) and SVZ (**F**) of C57BL/6 mice. The data are presented as the mean±SD of three independent experiments. Statistical significance was determined using unpaired *t*-tests. *$p < 0.05$, **$p < 0.01$, as compared with the Ctrl.

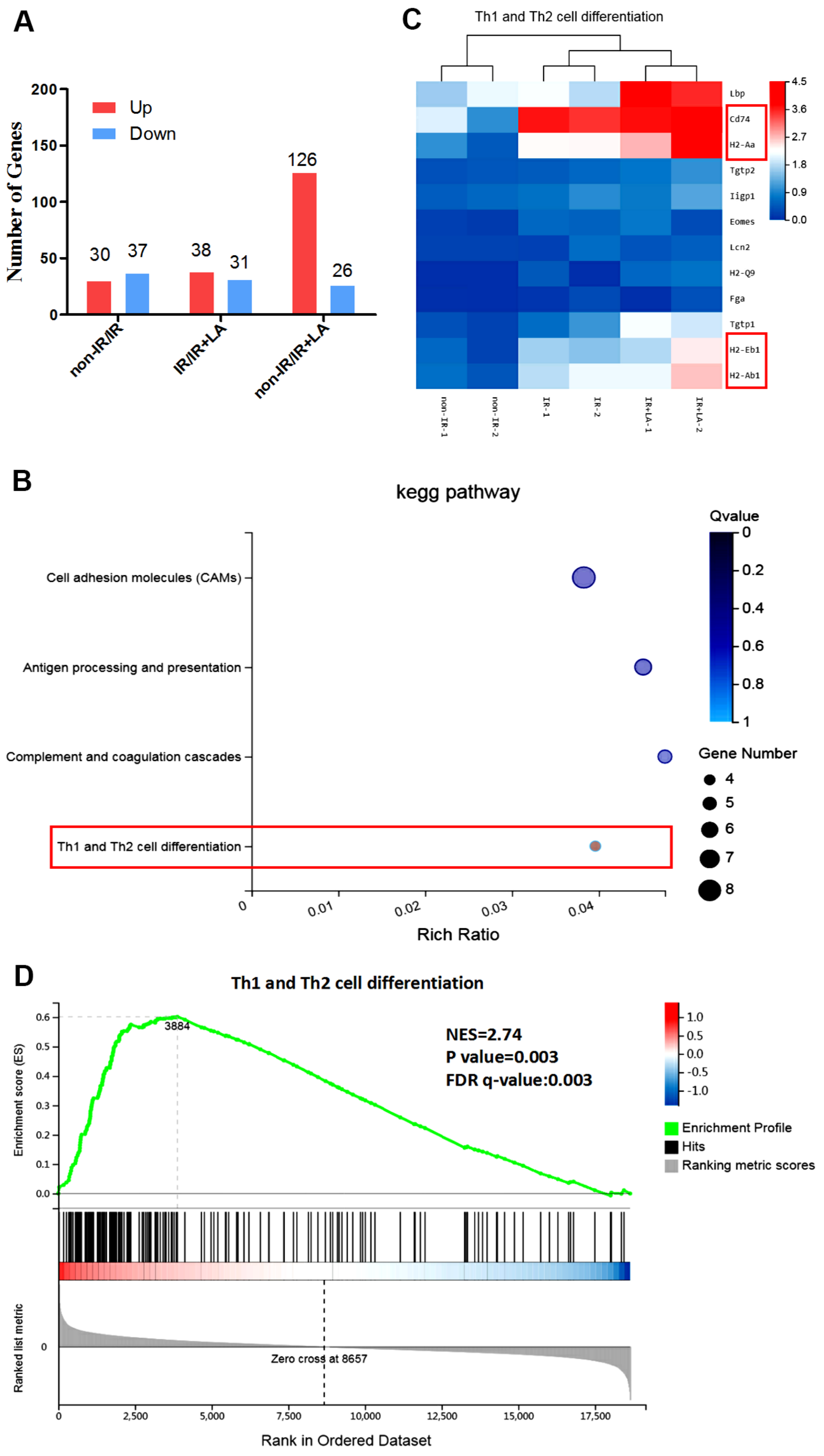

Supplementary Figure 3. Exploring the mechanism by which LA regulates the immune system by performing RNA-seq on the hippocampus of B-NDG mice. (**A**) The differentially expressed genes between non-IR and IR PBS-treated mice, between non-IR PBS-treated mice and IR LA-treated (IR+LA) mice, and between IR PBS-treated mice and IR+LA mice. (**B**) A bubble diagram showing the Th1- and Th2-cell differentiation signaling pathway regulated by LA treatment. (**C**) Heatmap showing the differentially expressed genes related to the Th1- and Th2-cell differentiation signaling pathway. (**D**) Enrichment plots of gene expression signatures for Th1- and Th2-cell differentiation. Barcode plot indicates the positions of genes in each gene-set. NES, normalized enrichment score. The data are presented as the mean±SD of three independent experiments. Statistical significance was determined using unpaired *t*-tests.*$p < 0.05$, **$p < 0.01$, as compared with the control.

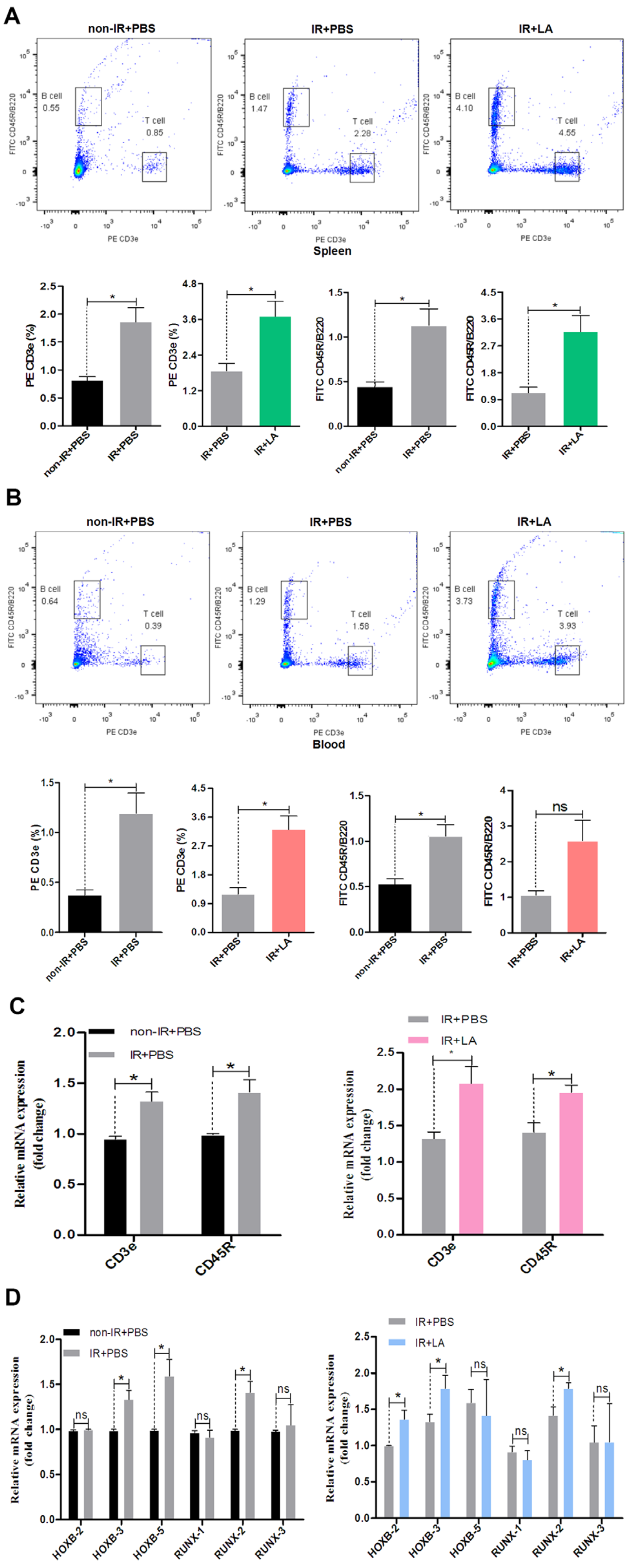

Supplementary Figure 4. The effect of LA on the *in vitro* proliferation of lymphocytes from B-NDG mice. (**A**, **B**) Proliferation of LA-treated or PBS-treated *in vitro* cultured T and B cells from the spleen (**A**) or whole blood (**B**) of IR B-NDG mice. (**C**) The relative mRNA expression of CD3e and CD45R. (**D**) Relative mRNA expression of genes that might regulate lymphocyte proliferation. The data are presented as the mean±SD of three independent experiments. Statistical significance was determined using unpaired *t*-tests.*$p < 0.05$, **$p < 0.01$, as compared with the Ctrl.
